# MoS_2_ memristor with photoresistive switching

**DOI:** 10.1038/srep31224

**Published:** 2016-08-05

**Authors:** Wei Wang, Gennady N. Panin, Xiao Fu, Lei Zhang, P. Ilanchezhiyan, Vasiliy O. Pelenovich, Dejun Fu, Tae Won Kang

**Affiliations:** 1 Key Laboratory of Artificial Micro- and Nano-Materials of Ministry of Education and School of Physics and Technology, Wuhan University, Wuhan 430072, China; 2 Department of Physics, Quantum-functional Semiconductor Research Center, Dongguk University, Seoul 100-715, Republic of Korea; 3 Institute of Microelectronics Technology, RAS, Chernogolovka, Moscow district, 142432, Russia; 4 Hubei Collaborative Innovation Center for Advanced Organic Chemical Materials, Faculty of Materials Science & Engineering, Hubei University, Wuhan 430062, China

## Abstract

A MoS_2_ nanosphere memristor with lateral gold electrodes was found to show photoresistive switching. The new device can be controlled by the polarization of nanospheres, which causes resistance switching in an electric field in the dark or under white light illumination. The polarization charge allows to change the switching voltage of the photomemristor, providing its multi-level operation. The device, polarized at a voltage 6 V, switches abruptly from a high resistance state (HRS_L6_) to a low resistance state (LRS_L6_) with the On/Off resistance ratio of about 10 under white light and smooth in the dark. Analysis of device conductivity in different resistive states indicates that its resistive state could be changed by the modulation of the charge in an electric field in the dark or under light, resulting in the formation/disruption of filaments with high conductivity. A MoS_2_ photomemristor has great potential as a multifunctional device designed by using cost-effective fabrication techniques.

A resistive switching phenomenon is of great interest due to its potential for the next generation nonvolatile resistance random access memory (RRAM), which has a low power dissipation, high speed of write/erase, good endurance, and scalability^1^,^2^,^3^. Switching the resistance in the electric field has been observed in various materials, such as graphene oxide^4^,^5^,^6^,^7^, amorphous carbon^8^, transition-metal oxide^3^,^9^,^10^,^11^,^12^,^13^, perovskites^14^,^15^,^16^, chalcogenides^17^,^18^,^19^,^20^,^21^, ferroelectrics^22^, and can be described as the drift of ions through the formation/rupture of conducting filaments^4^,^5^,^6^,^7^,^8^,^9^,^10^,^11^,^12^,^13^,^18^,^21^, trapping/detrapping of charge carriers^15^,^17^,^18^,^23^, or Fowler-Nowler tunneling^16^.

Molybdenum disulfide (MoS_2_), as a semiconducting analogue of graphene, with an indirect band gap of ~1.2 eV for the bulk material and with a direct gap of ~1.8 eV for the monolayer^24^ has tremendous potential for both electronic and optical applications. MoS_2_ is composed of stacked planes of covalently bonded S and Mo atoms with spacing of ~6.5 Å, which are weakly bound to each other by van der Waals forces. MoS_2_ in the form of flat flakes or nanospheres can be obtained by using the hydrothermal process and are uniformly distributed on a substrate using drop casting, vacuum filtration, Langmuir-Blodgett deposition, or spin coating processes^7^,^25^,^26^,^27^,^28^,^29^,^30^. The resistive switching in MoS_2_ structures in both vertical and lateral geometry has been reported^18^,^19^,^20^. In the case of 2 H MoS_2_ nanospheres consisting of monolayers, the polarization phenomenon can control the switching resistance^27^. Furthermore, nanosphere lattice, as reported^31^ can affect the electronic band gap, while the optical spectrum of nanoparticles can be changed in an electric field^32^,^33^ or under light^34^,^35^. Photodetectors constructed, for example, using ZnO spheres as building blocks demonstrate a high and fast photoresponse^36^.

In this article, we report a MoS_2_ nanosphere photomemristor obtained from the solution using a simple hydrothermal method. The memristor, through the charge polarization of nanospheres, can be switched by an electric field in the dark or under illumination with white light. Modulation of charge in an electric field by photons controlled switching of the photomemristor, providing multi-level resistance operation with great potential for advanced multi-functional non-volatile memories.

## Results and Discussion

**Figure 1:**
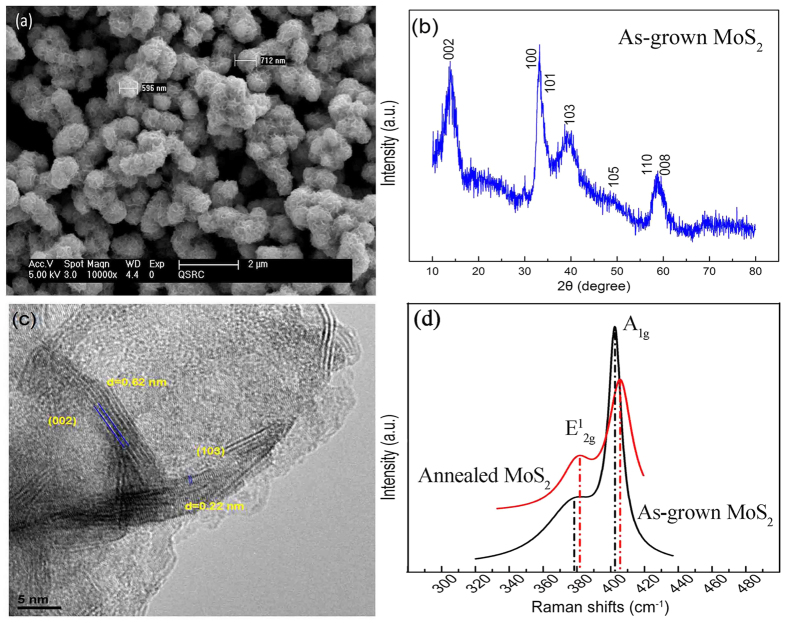
MoS_2_ nanospheres synthesized by using a hydrothermal technique. (**a**) An SEM image of nanospheres with diameters ranging from 550 to 750 nm after annealing. (**b**) XRD spectrum of as-grown MoS_2_ nanospheres. The diffraction peaks at 2θ = 14.1, 39.5, 49.4, 32.9, 33.8, 58.4 and 59.2 deg. correspond to the (002), (103), (105), (100), (101), (110), and (008) planes of the hexagonal MoS_2_ phase, respectively (JCPDS 75-1539). (**c**) A typical HRTEM image of the nanospheres indicating interlayer distances of 0.62 and 0.22 nm, which are indexed (002) and (103) planes of 2H-MoS_2_. (**d**) Raman spectra of as-grown and annealed MoS_2_ nanospheres. The positions of peaks were determined by using Gaussian fitting (Supplementary Information, Fig. S1). The fitted peaks at 375.5 and 401.3 cm^−1^ are characteristic for as-grown sample, but 383.5 and 406.2 cm^−1^ for the sample annealed in the H_2_/N_2_ atmosphere. The energy difference ΔE between E^1^_2_ _g_ and A_1_ _g_ modes corresponds to ~24 and ~22.7 cm^−1^ for 3–4 layers of MoS_2_ as-grown and annealed, respectively.

The MoS_2_ nanospheres synthesized using the hydrothermal method (see Methods) were 550~750 nm in diameter (Fig. 1(a)) with well-defined XRD spectrum (Fig. 1(b)). The diffraction peaks at 2θ = 14.1, 39.5, 49.4, 32.9, 33.8, 58.4 and 59.2 deg. correspond to the (002), (103), (105), (100), (101), (110), and (008) planes of the hexagonal MoS_2_ phase, respectively (JCPDS 75–1539)^37^,^38^,^39^,^40^. The (002) peak indicates that MoS_2_ grows with well-stacked layers along the (002) direction. Figure 1(c) shows a typical HRTEM image of the nanospheres. MoS_2_ interlayer distances of 0.62 and 0.22 nm are indexed (002) and (103) planes of 2H-MoS_2_, which agree well with the results of XRD measurements. Raman spectra of MoS_2_ nanospheres are shown in Fig. 1(d). The peaks at 375.5 and 401.3 cm^−1^ are characteristic for as-grown sample, but they are at 383.5 and 406.2 cm^−1^ for the sample annealed in the H_2_/N_2_ atmosphere. They correspond to E^1^_2_ _g_ and A_1_ _g_ vibrations of MoS_2_ atoms in plane and out of plane^38^,^39^,^41^. The shift of Raman peaks indicates an increase in van der Waals interaction between adjacent layers after annealing^32^. The energy difference ΔE between E^1^_2_ _g_ and A_1_ _g_ modes corresponds to ~24 and ~22.7 cm^−1^ for 3–4 layers of MoS_2_ as-grown and annealed, respectively. This is in good agreement with the HRTEM measurements (Fig. 1(c)).

**Figure 2:**
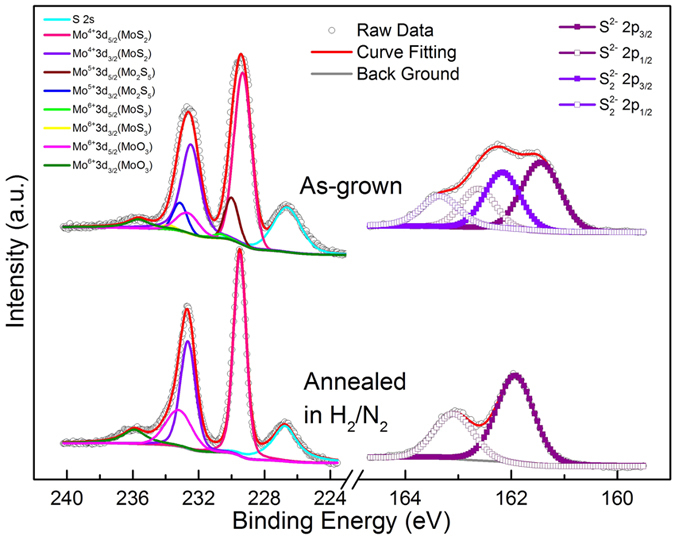
X-ray photoelectron spectra of the as-grown and annealed MoS_2_ nanostructures for Mo 3d, S 2s and S 2p core levels showing the MoS_2_ chemical states. For fitting of the peaks two doublets with area ratio of 1:2 or 2:3 and splitting of 1.18 eV or 3.13 eV were used for S 2 p and Mo 3 d, respectively. For as-grown nanospheres, Mo 3d_5/2_ peak comprises of four peaks centered at 229.32, 230.0, 231.01, and 232.65 eV, which correspond to MoS_2_, Mo_2_S_5_, MoS_3_ (Mo(S_2_)^2−^S^2−^)), and MoO_3_, respectively. S 2p_3/2_ peak is divided into two peaks centered at 161.44 and 162.16 eV, which correspond to MoS_2_ and intermediate product of Mo_2_S_5_ and MoS_3_. The intermediate phases are reduced in a stream H_2_/N_2_ and transferred to MoS_2_ phase in annealing process. The S 2p_3/2_ shows one peak at 161.92 eV.

X-ray photoelectron spectra of MoS_2_ nanospheres are shown in Fig. 2. For as-grown nanospheres, Mo 3d_5/2_ peak comprises of four peaks centered at 229.32, 230.0, 231.01, and 232.65 eV, which correspond to MoS_2_, Mo_2_S_5_, MoS_3_ (Mo(S_2_)^2−^S^2−^)), and MoO_3_, respectively (Table 1). S 2p_3/2_ peak is divided into two peaks centered at 161.44 and 162.16 eV, which correspond to MoS_2_ and intermediate product of Mo_2_S_5_ and MoS_3_, possibly due to incomplete reduction during the hydrothermal process^42^,^43^,^44^,^45^,^46^. After annealing, Mo 3d_5/2_ peak is divided into two peaks centered at 229.49 and 233.19 eV, which correspond to S-Mo-S and O-Mo-O, respectively. The S 2p_3/2_ peak shows only one peak at 161.19 eV (Table 1). It indicates that the Mo^4+^ state is predominant in annealed samples, and the intermediate phases of Mo_2_S_5_ and MoS_3_ are reduced in a stream H_2_/N_2_ and transferred to MoS_2_ phase. However, small quantities of MoO_3_ is present in the grown and annealed samples, possibly due to oxidation of MoS_2_ during the hydrothermal process. Finding of the S 2** **s peak at 226.61 eV and atomic S/Mo ratio of about 2 for the annealed sample (Table 1) indicates that the MoS_2_ nanospheres are close to the stoichiometric composition.

**Figure 3:**
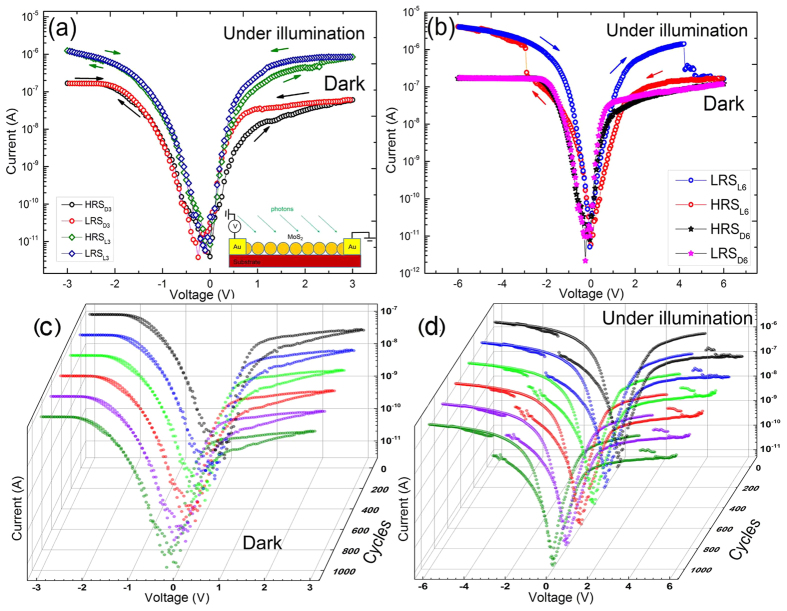
The current-voltage (I-V) chracteristics of the Au/MoS_2_ nanospheres/Au memristor in dark or under white light in a logarithmic scale at room temperature. Light with spectral maxima at 2.7 and 1.8 eV was used as a white light source. Schematic of the device in the inset of Fig. 3(a). Arrows on the I/V curves indicate the sweep direction of the voltage. (**a**) I–V curves after sweeping the poling voltage 3 V at a constant rate (0.05 V s^−1^). The device polarized at 3 V is smoothly switched from HRS_L3_ to LRS_L3_ under a white light and from HRS_D3_ to LRS_D3_ in the dark with a ratio of On/Off resistances about 2 and 4 at 1.2 V and 0.7 V, respectively. (**b**) I–V curves after sweeping the poling voltage 6 V at a constant rate (0.05 V s^−1^). The device polarized at 6 V shows a sharp resistive switching under white light from HRS_L6_ to LRS_L6_ at −2.9 V with a ratio of On/Off resistances about 10 and a smooth switch from HRS_D6_ to LRS_D6_ in the dark with a ratio of On/Off resistances about 3 at 0.7 V. (**c**) Memristive characteristics of the device in the dark after multi-cycles. (**d**) Memristive characteristics of the device under white light after multi-cycles.

Figure 3 shows the current-voltage (I–V) curves of the Au/MoS_2_ nanospheres/Au structure after sweeping the poling voltage (3 and 6 V) at a constant rate (0.05 V s^−1^). A schematic image of the device is shown in Inset of Fig. 3(a), and Supplementary Information, Fig. S5. Nonlinear characteristics of the device with a marked hysteresis indicates the memristive behaviour. Furthermore, the memristive structure showed a high photoresponse to white light (spectral maxima at 2.7 and 1.8 eV), as the increase in current 1500% and 2000% at a voltage of 3 and 6 V, respectively. When the device is polarized at 3 V smooth switching occurs from HRS_L3_ to LRS_L3_ under a white light, and from HRS_D3_ to LRS_D3_ in the dark with a ratio of On/Off resistances about 2 and 4 at 1.2 V and 0.7 V, respectively (Fig. 3(a)). At higher poling voltage (6 V), the device shows a sharp resistive switching under white light from HRS_L6_ to LRS_L6_ at −2.9 V with a ratio of On/Off resistances about 10 and a smooth switch from HRS_D6_ to LRS_D6_ in the dark with a ratio of On/Off resistances about 3 at 0.7 V (Fig. 3(b)). The SET operation from OFF to ON state represents a “writing” process. When the applied voltage runs from 0 to the positive voltage (4.2 V), the structure reverts to the HRS_L6_. The operation RESET from the ON to the OFF position acts as a function of erasing. Memristive behavior of the device in the dark and under white light is well reproduced in the process of iteration to 1000 cycles with excellent stability of the states (Fig. 3(c,d) and Supplementary Information, Fig. S3). It indicates that the multi-level resistive switching of the memristor nanostructure can be obtained and controlled by an electric field in the dark and under white light.

**Figure 4:**
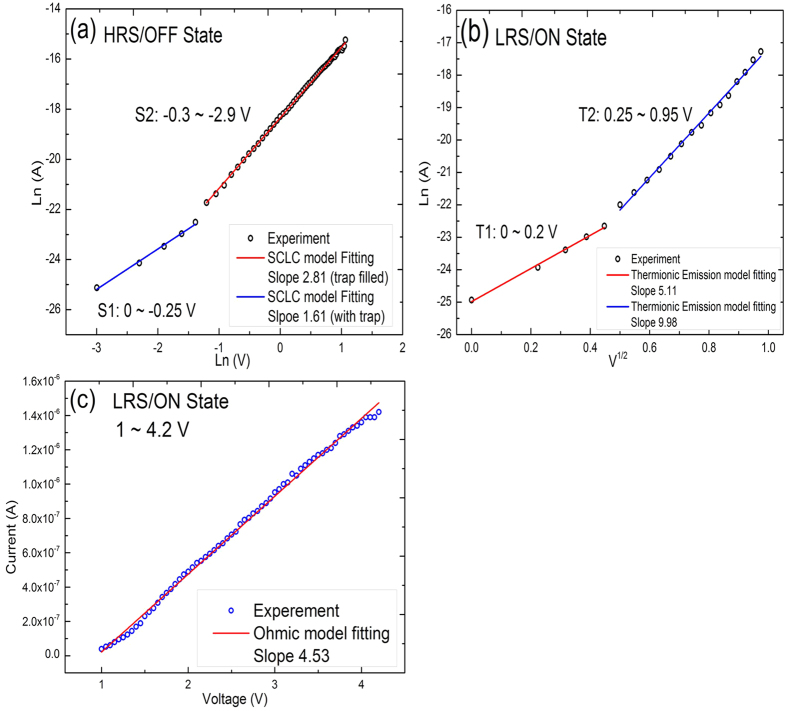
Analysis of the mechanism of conductivity in the resistive states of the memristor polarized at 6 V under white light. (**a**) I/V characteristics in the HRS/OFF state. Two linear relations used to fit the logarithmic plot of current vs. voltage from 0 to −0.25 V (denoted as S1) and from −0.25 to −2.9 V (denoted as S2) given the slopes of 1.61 and 2.81, respectively. Resistance behavior changes from trap-unfilled (S1) to trap-filled (S2) described by the SCLC which contributes to charge emission. (**b**) I/V characteristics in the LRS/ON state when the voltage is less than 1 V. The plots of Ln(I) vs. V^1/2^ from 0 to 0.2 V (denoted as T1) and 0.25 to 0.95 V (denoted as T2) are well fitted to straight lines with a slope of 5.11 and 9.98 for T1 and T2, respectively, by using the thermionic emission model. (**c**) I/V characteristics in the LRS/ON state when the voltage exceeds 1 V. The I–V curve obeys the ohmic conduction model with a slope of 4.53, demonstrating that good conductive channels are established in the ON state.

The current-voltage characteristics of the memristor were investigated using the thermionic emission (TE) and the space charge limited current (SCLC) models^4^,^5^,^6^,^8^,^9^,^10^,^11^,^12^,^13^,^14^,^15^,^16^,^17^,^21^,^22^ to clarify the mechanism of conduction in the resistive states (Fig. 4, Supplementary Information, Fig. S4). In the HRS/OFF state, two linear relations were used to fit the logarithmic plot of current vs. voltage from 0 to −0.25 V (denoted as S1) and from −0.25 to −2.9 V (denoted as S2) given the slopes of 1.61 and 2.81, respectively (Fig. 4(a)). The conduction mechanism in HRS is described by the SCLC model, which is dominant in the carrier transport process of the OFF state and originates from the charge trapping and detrapping by MoS_2_ nanosphere interfaces. Defects at MoS_2_ interfaces serve as charge traps, assisting the nanosphere polarization. When negative voltage is applied to the structure, the injected electrons are captured by the defects and fill the traps. Resistance behavior changes from trap-unfilled (S1) to trap-filled (S2) which describes the SCLC contribution to charge emission. In the LRS/ON state, when the voltage is less than 1 V, the plots of Ln(I) vs. V^1/2^ from 0 to 0.2 V (denoted as T1) and 0.25 to 0.95 V (denoted as T2) are well fitted by using the thermionic emission model to straight lines with a slope of 5.11 and 9.98 for T1 and T2, respectively (Fig. 4b). When the voltage exceeds 1 V, the I–V curve obeys the ohmic behavior with a slope of 4.53, demonstrating that good conductive channels are established in the ON state (Fig. 4(c)). Formation of the conduction channels along the boundaries of polarized nanospheres, which are interrupted by a reverse bias could be the driving force behind the resistance switching. An electric field can control the polarization of the nanospheres and the shift of the polarization domain in the dark^27^ and under the light through the additional charge generating by photons with energies that exceed the band gap of MoS_2_ (1.5–1.9 eV). Electrons and holes are separated at nanosphere interfaces, affecting the collective polarization of the nanospheres and the interface barrier height.

**Figure 5:**
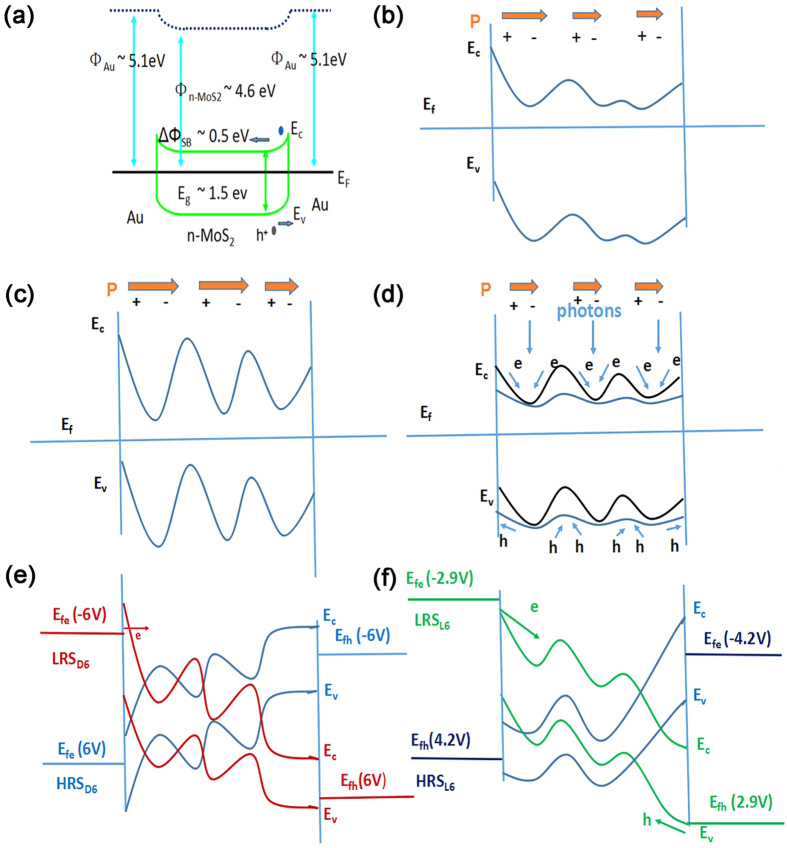
Schematic band diagrams of Au/MoS_2_ nanospheres/Au structure polarized in an external electric field in the dark or under white light. (**a**) The initial nanosphere structure. The band alignments are based on work functions of n-type MoS_2_ (Φ_n-MoS2_ = 4.6 eV) and Au (Φ_Au_ = 5.1 eV). (**b**) The nanosphere structure with barriers on polarized nanosphere interfaces at 3 V in the dark. (**c**) The nanosphere structure with higher barriers on more polarized nanosphere interfaces at 6 V in the dark. (**d**) Polarized nanosphere structure under white light excitation at 3 V (blue) and 6 V (black). (**e**) The polarized nanostructure at the SET and RESET voltages −6 V and 6 V in the dark. Electron tunneling through the barrier in the dark at −6 V forms LRS_D6_. At an applied voltage of 6 V the memristor sets a high barrier at the interface of polarized nanospheres and switches back to HRS_D6._ (**f**) The polarized nanostructure at the SET and RESET voltages −2.9 V and 4.2 V under white light. The memristor is switched at −2.9 V from HRS_L6_ to LRS_L6_ (SET operation). A reversely applied electric field leads to the recovery of the interface barrier height, hindering electron tunneling. The conductive channel is destroyed at 4.2 V, and the memristor is switched to HRS_L6_ (RESET). The valence (E_v_) and conductance (E_c_) bands of the MoS_2_ nanospheres in LRS_D6_ and HRS_D6_ in the dark, in red and blue (**e**), and in LRS_L6_ and HRS_L6_ under white light, using green and blue colors (**f**), respectively. E_fe_ and E_fh_ are the positions of the Fermi level in the structure for electrons and holes.

Figure 5 shows the schematic band diagram of the memristor structure. The band alignments (Fig. 5(a)) are based on reported work functions of n-type MoS_2_ (Φ_n-MoS2_ = 4.6 eV), and Au (Φ_Au_ = 5.1 eV)^47^. Two Schottky barriers (ΔΦ_SB_ = Φ_Au_ − Φ_n-MoS2_ = 0.5 eV) and Shottky-like double barriers of nanosphere interfaces can separate photo-generated electron-hole pairs which produce a photocurrent. Poling the structure (Fig. 5 (b,c)) forms the interface barriers and polarization **P** of nanospheres (brown arrows). White light exposure to the nanospheres decreases the interface barrier hight (Fig. 5 (d,f)). The nanospheres are likely to play a significant role in the memristor behavior. An external electric field charges the nanospheres and polarizes them, forming the percolation conduction channels along the interfaces. The charge injection by light contributes to nanosphere polarization over a large area between the lateral electrodes and effect on switching characteristics. The injection of electrons into nanospheres through tunneling process and photogeneration in a high electric field decreases the barrier height at the interface and forms a conductive channel. The memristor is switched at −2.9 V under white light from HRS_L6_ to LRS_L6_ (SET operation) (Fig. 5(f)). A reversely applied electric field leads to opposite charge injection and recovery of the interface barrier height, hindering electron tunneling. The conductive channel is destroyed at 4.2 V (Fig. 5(f)), and the memristor is switched to HRS_L6_ (RESET). SET operation in the dark results in a higher resistance state due to a higher barrier at the interface. Electron tunneling through the barrier at −6 V forms LRS_D6_ (Fig. 5(e)). At an applied voltage of 6 V the memristor sets a high barrier at the interface of oppositely polarized nanospheres and switches back to HRS_D6_ (Fig. 5e). It should be noted that a high density of point defects may affect the behavior of the memristive device, especially on the nanoscale. Resistive switching in the semiconductor 2 H MoS_2_ phase with grain boundaries in the presence of a high density of sulfur vacancies was reported^19^. However, MoS_2_ nanospheres used in our experiments were close to the stoichiometric composition (Table 1). The ion drift at a low concentration of point defects in the semi-insulating nanospheres hardly could control the resistive switching on the observed micrometer scale. But the nanospheres can be charged and polarized by an external electric field, as previously reported^27^,^48^, changing their resistance because of the motion of the collective polarization domain. It is worth noting that a T ferroelectric phase, recently discovered in MoS_2_^20^,^49^ can also cause non-linearity of current-voltage characteristics and the resistive switching. Distortion of the 1 T MoS_2_ lattice under an external electric field can cause resistance to change. However, the 1 T phase can be easily converted into a 2 H phase at moderate annealing temperatures (above 200 °C)^50^. In addition, the T phase is highly conductive and thus has a low photosensitivity.

**Figure 6:**
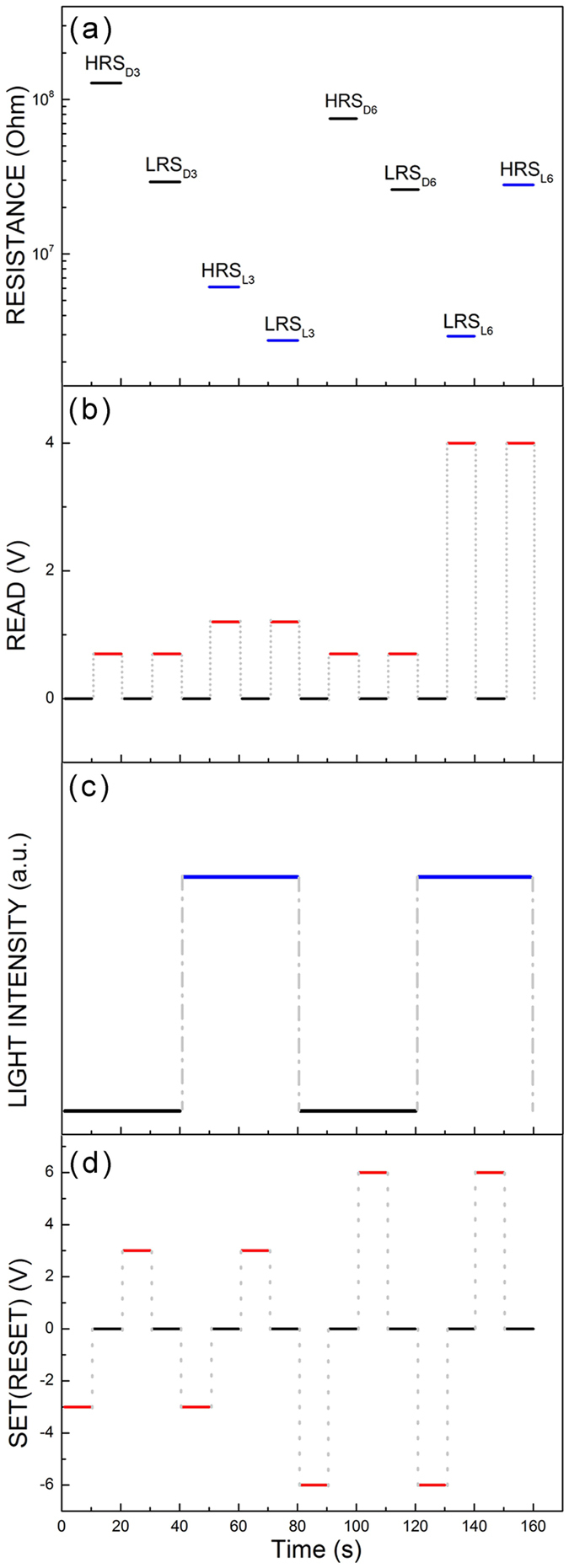
Operation of the MoS_2_ memristor polarized at different voltages in the dark or under white light excitation. (**a**) High and low resistance states obtained by using the SET/RESET operations at −3 V/+3 V and −6 V/+6 V in the dark (HRS_D3_, LRS_D3_ and HRS_D6_, LRS_D6_) and under white light (HRS_L3_, LRS_L3_, LRS_L6_, and HRS_L6_). (**b**) Pulse voltage reading chart. The resistive states are read out at 0.7 V (HRS_D3_, LRS_D3_, HRS_D6_, and LRS_D6_), 1.2 V (HRS_L3_ and LRS_L3_) and 4 V (LRS_L6_ and HRS_L6_) in the dark (_D_) or under white light excitation (_L_). (**c**) The diagram of excitation by white light pulses. SET/RESET, and the READ operation is controlled by pulses of light off (black) (HRS_D3_, LRS_D3_, HRS_D6_ and LRS_D6_) and on (blue) (HRS_L3_, LRS_L3_, HRS_L6_ and LRS_L6_). The memristor polarized at 3 V exhibits the four states, which are read as HRS_D3_, LRS_D3_, HRS_L3_ and LRS_L3_ while the memristor polarized at 6 V shows the other four states HRS_D6_, LRS_D6_, HRS_L6_ and LRS_L6_ which can be read in the dark or under white light.

The charge modulation by photons allows control of the switching voltage of the 2 H MoS_2_ photomemristor, providing the multilevel resistive switching operation. The multilevel switching of resistance of the polarized device poled at different voltages under white light or in the dark was observed in our experiment (Fig. 3). The natural application for resistive switching is nonvolatile resistive random access memory, however their dynamical nonlinear behavior also suggests that it could be used to develop alternative logic architectures. Figure 6 demonstrates the resistance states formed after the SET/RESET operations of the MoS_2_ memristor polarized at voltages of 3 V and 6 V in the dark or under light excitation. The memristor polarized at 3 V in the dark or under white light demonstrates the four states, which can be read at a voltage of 0.7 V (HRS_D3_ and LRS_D3_) and 1.2 V (HRS_L3_ and LRS_L3_) in the dark or under light (Fig. 6(a)). The memristor polarization at 6 V in the dark or light led to the formation of the other four states, which are read at a voltage of 0.7 V (HRS_D6_ and LRS_D6_) and 4 V (HRS_L6_ and LRS_L6_) in the dark or light (Fig. 6(a)). All of these states are well controlled electrically at room temperature with the applied voltage and light excitation as it is confirmed by the iterative operation of the memristor under different conditions of writing and reading (Fig. 3(с and Supplementary Information, Fig. S3). Polarization of nanospheres in the photomemristor using pulses of electric field and light creates multi-level resistance states. Analysis of conductivity in the resistance states indicates that charging and nanosphere polarization modulated by light could generate and interrupt conductive filaments to switch resistance. Reducing the gap between the electrodes in a planar geometry or vertical, can greatly minimize the operating voltage of the device. Modulation of the barrier height at the interfaces of nanospheres in an external electric field by light appears to be highly promising for high-speed optoelectronics, since the polarization process is much faster than the ion drift^36^.

In conclusion, we have demonstrated a MoS_2_ nanosphere-based photomemristor. The resistance of the device can be controlled by the polarization of nanospheres and switched by an electric field in the dark and under white light. The charge modulation by photons allows to control the switching characteristics of the photomemristor, providing the multi-level resistance switching operation. The memristor polarized at different voltages demonstrates several resistance states readable under white light and in the dark. We believe that the photomemristor has great potential as a new multifunctional device made by simple and inexpensive methods.

## Methods

### Fabrication of the Au/MoS_2_/Au structure

The MoS_2_ nanospheres were grown by using the hydrothermal technique. Sodium molybdate (Na_2_MoO_4_·2H_2_O, 0.03 g) and thioacetamide (CH_3_CSNH_2_, 0.06 g) were dissolved in 20 mL distilled water. After stirring for 1 hour, the solution was transferred into a 100 mL Teflon-lined stainless steel autoclave and heated at 220 °C for 24 hours. After cooling down to 22 °C. the black precipitates of MoS_2_ were first retrieved from the solution, collected, and washed with acetone, methanol, distilled water, and absolute ethanol for five times, and dried in vacuum oven at 80 °C for 6 hours. Then, the dispersion of MoS_2_ nanospheres mixed with isopropyl alcohol (IPA) and was spin-coated on a Si substrate with 275-nm-thick SiO_2_ and baked at 200 °C for 15 minutes twice, followed by a rapid thermal annealing at 800 °C for 5 minutes in a stream of 200 sccm (H_2_/N_2_ = 2:3). Finally, 50-nm-thick Au electrodes with a width of 980 μm, length of 8.4 mm, and spacing of 226 μm (Fig. S5 in the Supporting Information) were fabricated sequentially via photolithography, e-beam evaporation and the lift-off method. After this the samples were annealed in a stream of 200 sccm (H_2_/N_2_ = 2:3) at 450 °C for 15 minutes.

### Characterizations

The crystal structure of the MoS_2_ sample was analyzed by X-ray diffraction techniques (XRD, Bruker D8 Advance) with a Cu K_α_ radiation (λ = 0.154 nm), high resolution transmission electron microscopy (HRTEM, JEOL JEM 2010), and a room temperature micro-Raman spectrometry (LabRAM HR800, He-Ne laser, 488 nm). The morphology and chemical composition of MoS_2_ were examined by a field emission scanning electron microscopy (FESEM, S4800) and X-ray photoelectron spectroscopy (XPS, Escalab 250Xi, Mg K_α_ excitation at 1253.6 eV, 150 W). The current-voltage (I–V) characteristics of fabricated Au/MoS_2_/Au structures were investigated at room temperature in ambient air in the dark and under white light excitation (spectral maxima at 2.7 and 1.8 eV) using a Keithley 4200 SCS semiconductor parameter analyzer with LabVIEW software in voltage sweeping mode.

## Additional Information

**How to cite this article**: Wang, W. *et al*. MoS_2_ memristor with photoresistive switching. *Sci. Rep.* **6**, 31224; doi: 10.1038/srep31224 (2016).

## Supplementary Material

Supplementary Information

## Figures and Tables

**Table 1:** The fitted Mo 3d and S 2p peak position and atomic percentages of as-grown and annealed MoS_2_ nanospheres.

Peak and indensity		Peak position (eV) (at.%)	
		As-grown	Annealed
Mo^4+^ 3d_5/2_	MoS_2_	229.32 (43.5)	229.49 (45.9)
Mo^4+^ 3d_3/2_		232.47 (29.5)	232.64 (29.8)
Mo^5+^ 3d_5/2_	Mo_2_S_5_	230.00 (8.6)	/
Mo^5+^ 3d_3/2_		233.13 (5.7)	/
Mo^6+^ 3d_5/2_	MoS_3_	231.01 (1.2)	/
Mo^6+^ 3d_3/2_		233.83 (0.8)	/
Mo^6+^ 3d_5/2_	MoO_3_	232.65 (6.5)	233.19 (16.3)
Mo^6+^ 3d_5/2_		235.65 (4.2)	235.89 (8.0.)
S^2−^2p_3/2_	MoS_2_	161.44 (36.2)	161.19 (66.7)
S^2−^2p_1/2_		162.62 (18.1)	163.08 (33.3)
S_2_^2−^2p_3/2_	/	162.16 (27.6)	/
S_2_^2−^2p_1/2_		163.34 (18.1)	/
S/Mo		1.19	2.12
