# Corrigendum: MoS_2_ memristor with photoresistive switching

**DOI:** 10.1038/srep33107

**Published:** 2016-11-14

**Authors:** Wei Wang, Gennady N. Panin, Xiao Fu, Lei Zhang, P. Ilanchezhiyan, Vasiliy O. Pelenovich, Dejun Fu, Tae Won Kang

Scientific Reports 6: Article number: 3122410.1038/srep31224; published online: 08 05 2016; updated: 11 14 2015

This Article contains errors in the Acknowledgements section.

“This work was supported by the National Natural Science Foundation of China under grant 11405117, by the National Research Foundation of Korea (NRF) grant funded by the Korea government (MSIP) (No. 2015-066177), and by International Program of the Ministry of Science and Technology of China under grant 2015DFR00720”.

should read:

“This work was supported by the National Research Foundation of Korea (NRF) grant funded by the Korea government (MSIP) (No. 2015-066177), International cooperation program managed by NRF (S-2015-A0173-00005), National Natural Science Foundation of China under grant 11405117, and International Program of the Ministry of Science and Technology of China under 2015DFR00720”.

