# Supplementary material for: MoS2 memristor with photoresistive switching
Source: Sci Rep. 2016 Aug 5;6:31224. doi: 10.1038/srep31224 (PMC4974562; doi:10.1038/srep31224)
Supplement: Supplementary Information [file srep31224-s1.doc]

Supplementary Information

**MoS_2_ memristor with photoresistive switching**

Wei Wang,^1,2^ Gennady N. Panin,^2,3,a)^ Xiao Fu,^2^ Lei Zhang,^4^ P. Ilanchezhiyan,^2^ Vasiliy O. Pelenovich,^1^ Dejun Fu,^1,b)^ Tae Won Kang^2^

^1^Key Laboratory of Artificial Micro- and Nano-Materials of Ministry of Education and School of Physics and Technology, Wuhan University, Wuhan 430072, China

^2^Department of Physics, Quantum-functional Semiconductor Research Center, Dongguk University, Seoul 100-715, Republic of Korea

^3^Institute of Microelectronics Technology, RAS, Chernogolovka, Moscow district, 142432, Russia

^4^Hubei Collaborative Innovation Center for Advanced Organic Chemical Materials, Faculty of Materials Science & Engineering, Hubei University, Wuhan 430062, China

**Corresponding Author**

^a)^E-mail:[g_panin@dongguk.edu](mailto:g_panin@dongguk.edu) (G. N. P.) ^b)^ E-mail: [djfu@whu.edu.cn](mailto:djfu@whu.edu.cn) (D. J. F.).


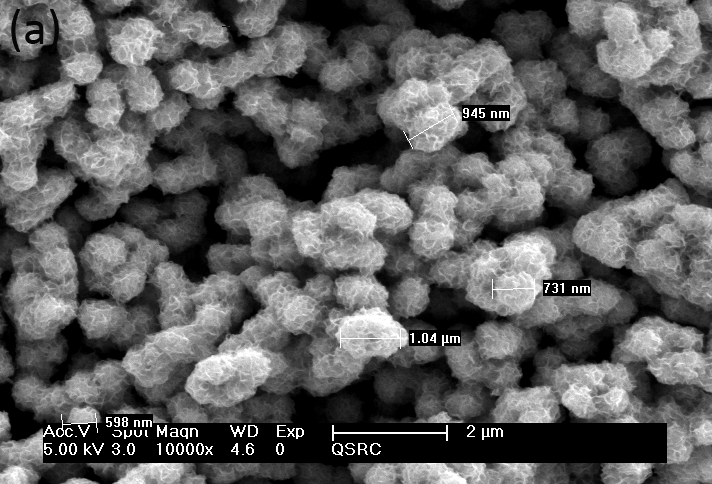

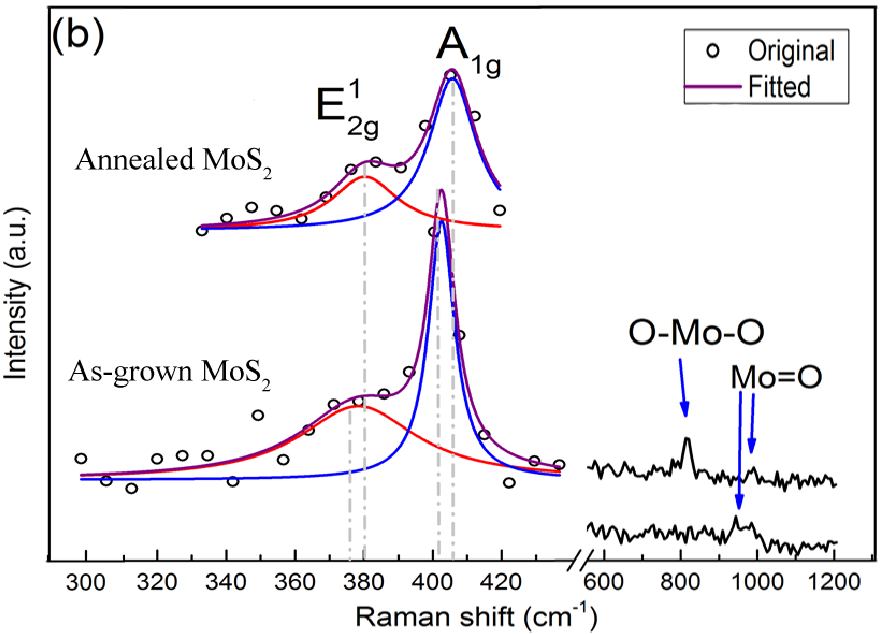


**Figure S1. (**a) An SEM image of as-grown MoS_2_ nanospheres and (b) Raman spectra of the as-grown and annealed MoS_2_ nanosphere samples.

The as-grown MoS_2_ nanospheres are in the range from 590 to 1010 nm in diameter (Figure S1. **(**a)). Typical Raman spectra of the MoS_2_ nanospheres synthesized by the hydrothermal method at 220 ºC for 24 hours and annealed in a stream of 200 sccm (H_2_/N_2_ = 2:3) at 800 ºC for 5 minutes with rapid thermal annealing process are shown in Figure S1b. The position of peaks were determined by using Gaussian fitting. The peaks at 375.5 and 401.3 cm^-1^ are a characteristic for the as-grown sample, but 383.5 and 406.2 cm^-1^ for the sample annealed in the H_2_/N_2_ atmosphere. They correspond to E^1^_2g_ and A_1g_ vibrations of MoS_2_ atoms in plane and out of plane, respectively. The energy difference ΔE between E^1^_2g_ and A_1g_ modes corresponds to ~24 and ~22.7 cm^-1^ for 3-4 layers of MoS_2_ as-grown and annealed, respectively. The peaks at 816 and 956 cm^-1^ belong to MoO_3_ with O-Mo-O and Mo=O stretching vibrations, respectively,^1,2^ which could be a result of partial oxidation during the hydrothermal process or measurement.^3,4^


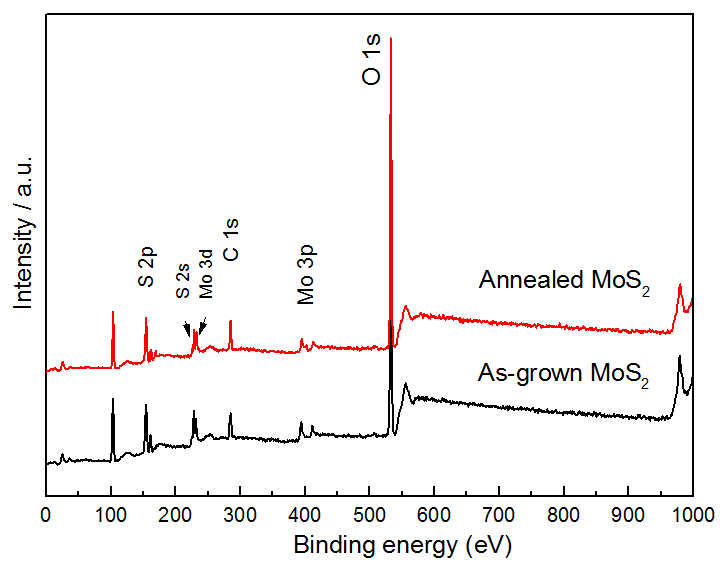


**Figure S2.** The X-ray photoelectron spectroscopy (XPS) spectrum, corresponding to the survey core levels for the as-grown and annealed MoS_2_.

The XPS spectrum for the as-grown sample is shown in Figure S2. It demonstrates several characteristic peaks of MoS_2_: S2p, S2s, Mo3d, Mo3p, which were used to determine the composition of the sample, and the ratio of Mo/S. The appearance of the peak of oxygen and carbon associated with contamination of the sample during the hydrothermal process or measurement.


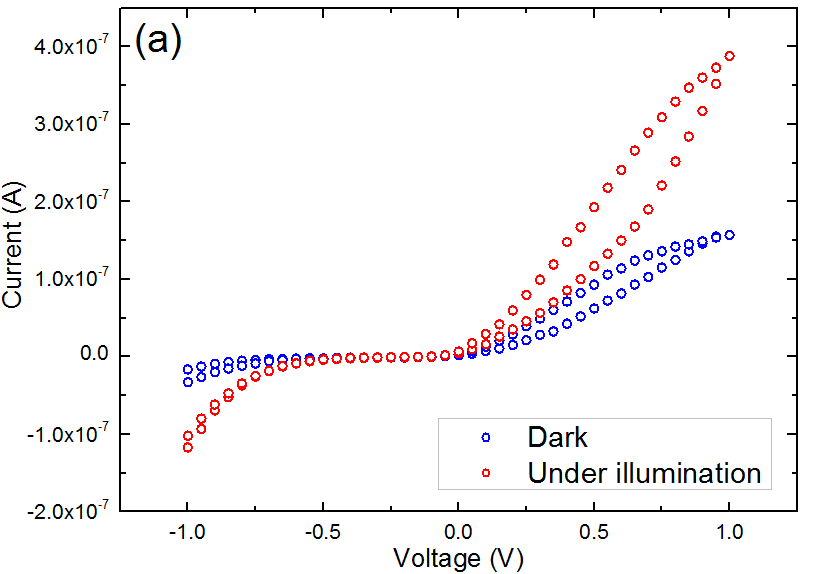

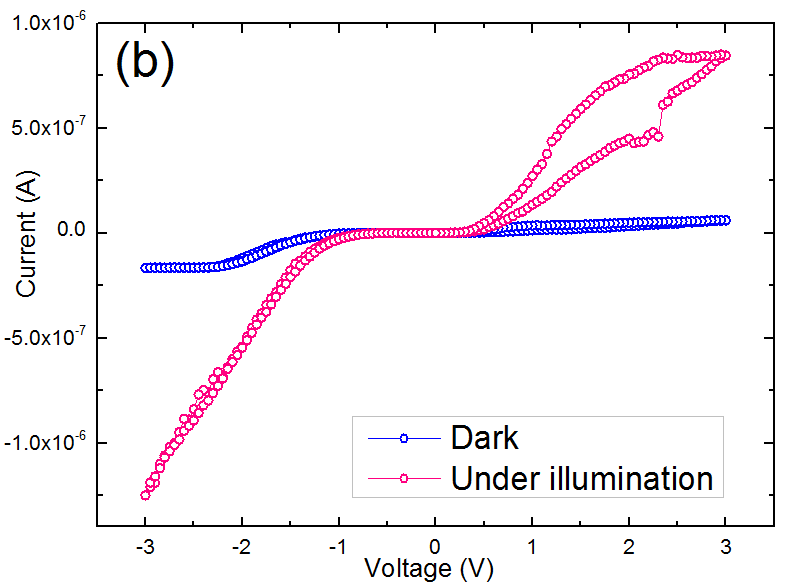

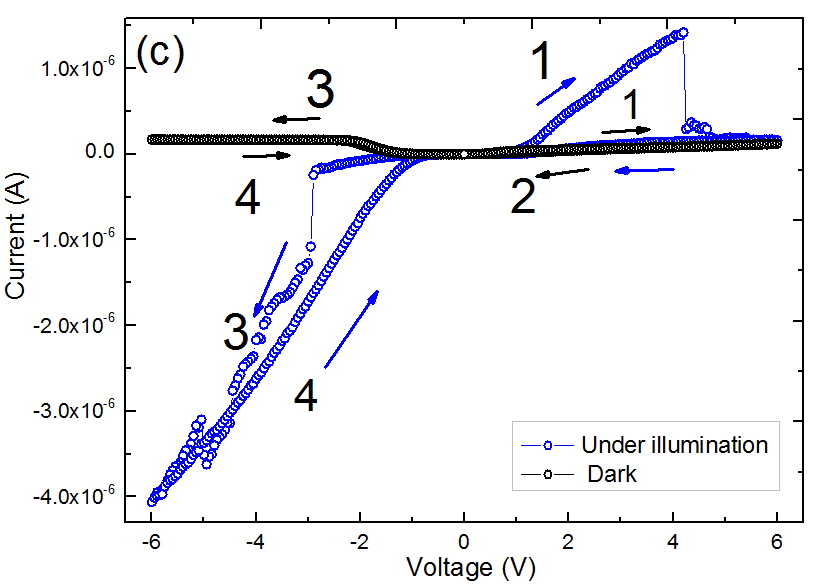

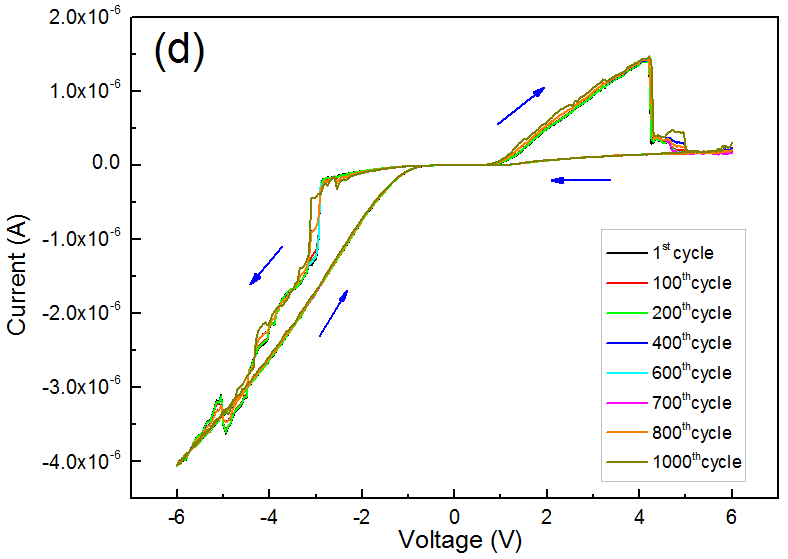


**Figur****e S3.** The current-voltage curves of the Au/MoS_2_ nanospheres/Au structure in dark or under white light in linear scale at room temperature at different sweep voltage ranges: a) from -1 to +1 V, b) from -3 to +3 V and c) from -6 to +6 V. d) Memristive device characteristics measured under white light are presented after every 100 or 200 cycles up to 1000 cycles. All curves are well reproducible with low dispersion and show a good stability after a series of switching cycles. The light with spectral maxima at 2.7 and 1.8 eV was used as a white light source. The arrows indicate the direction of the voltage sweep.

Current-voltage curves of the Au/MoS_2_/Au structure are non-linear and asymmetric, indicating non-ohmic contacts of Au/ MoS_2_ (Schottky barriers) and an inhomogeneous distribution of defects near the contacts. Clockwise direction of the hysteresis loops show that the electron trapping and polarization takes place in the MoS_2_ structures.^5-8^

To clarify the mechanism of conduction in resistive states (HRS and LRS) the current-voltage characteristics of the Au/MoS_2_/Au structures were investigated using the thermionic emission (TE) and the space-charge-limited current (SCLC) models, as well as an ohmic conduction model.

SCLC model:

$I\propto\frac{A\mu\varepsilon n}{n_{t}}(V^{2}/d^{3})$ (S1)

TE model:

(S2)

Ohmic model:

$I\propto V\exp(\frac{-\Delta E_{ae}}{kT})$ (S3)

where $I$,$V$, $A$, $T$, $k$, , $n_{t}$, $q$, $d$, $\Delta E_{ae}$, $\pi$, and $\varepsilon$ are the current, voltage, Richardson constant, ambient temperature, Boltzmann constant, Schottky energy barrier, concentration of trapped charge, electron charge, thickness of active layer, activation energy of an electron, ratio of circumference to diameter, and the electric constant, respectively.


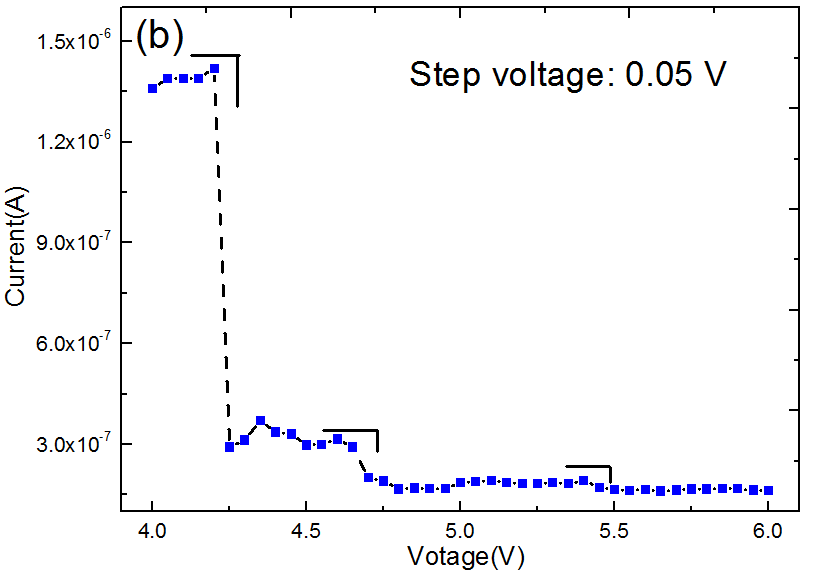

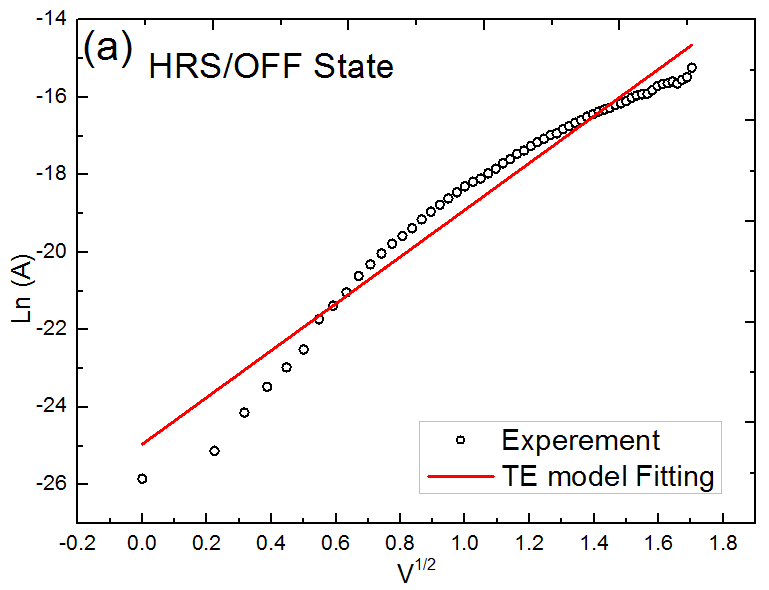


**Figure S4.** Analysis of the mechanism of conductivity in the high resistive state of the memristor polarized at 6V under white light based on the TE model (a) and a resistive switching of the device in the range of 4 to 6 V (b).

Figure S4 shows that I/V characteristics in the HRS/OFF state do not match the TE model (red line), which means that the conduction mechanism is different. The conduction mechanism in HRS is described by the SCLC model (Fig. 4(a)), which is dominant in the carrier transport process of the OFF state and originates from the charge trapping and detrapping by MoS_2_ nanosphere interfaces. The abrupt switching of current at 4.2 V (Fig S4 (b)) from the On state to Off state during the voltage sweep at a constant rate 0.05 V s^-1^, indicates that this operation is much faster than the sweep rate.


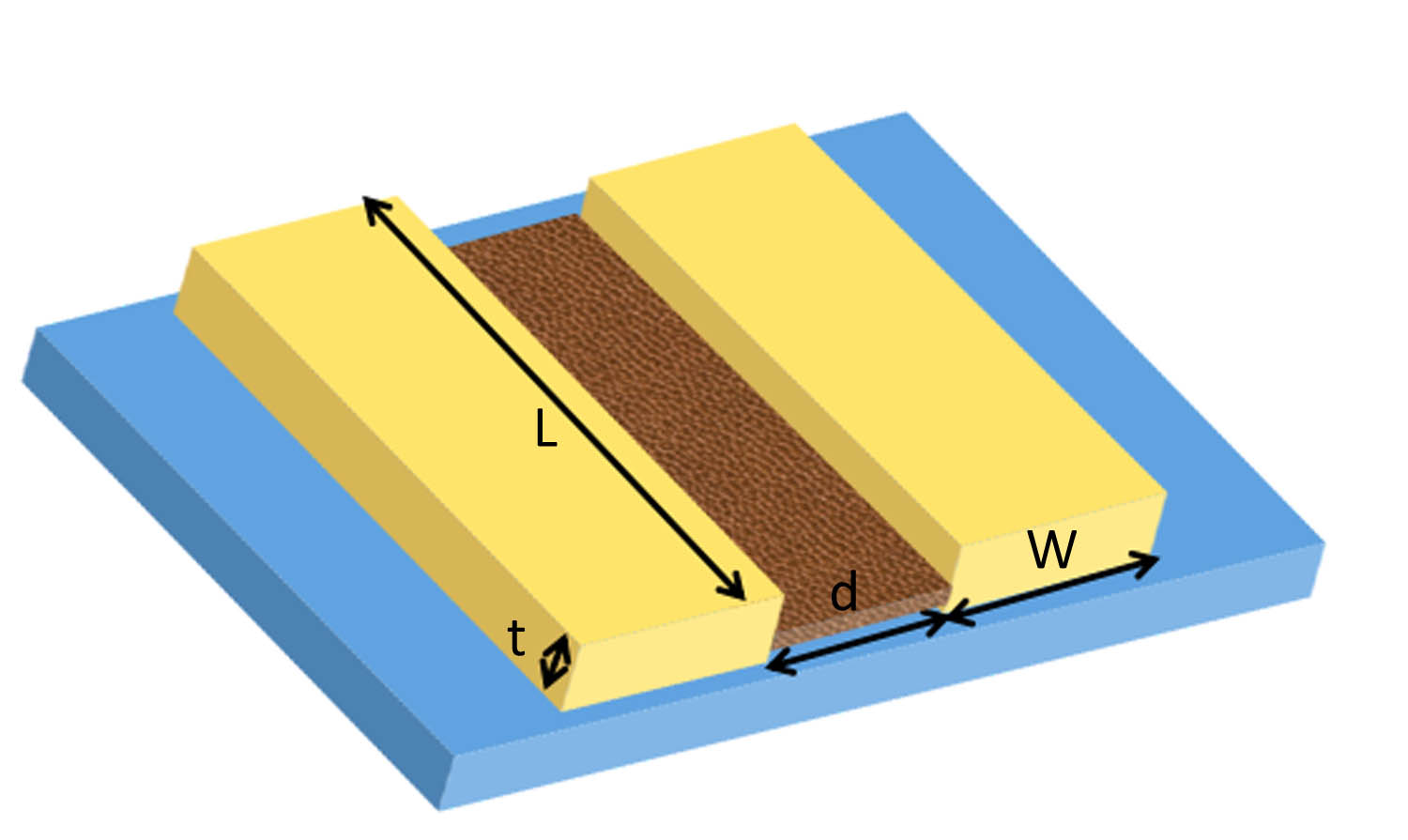


**Figure S5.** Schematic of memristor based on MoS_2_ nanospheres (brown) with Au electrodes (yellow) on a Si/SiO_2_ substrate (blue). The thickness (t), width (W), length (L) and spacing (d) of Au electrodes are 50 nm, 980 µm, 8.4 mm, and 226 µm, respectively.

The dispersion of MoS_2_ nanospheres mixed with IPA was spin-coated on a Si substrate with 275-nm-thick SiO_2_ and baked at 200 ºC for 15 minutes twice, followed by a rapidly thermal annealing at 800 ºC for 5 minutes in a stream of 200 sccm (H_2_/N_2_ = 2:3). 50-nm-thick Au electrodes with a width of 980 µm, length of 8.4 mm, and spacing of 226 µm (Figure S5) were fabricated sequentially via photolithography, e-beam evaporation and the lift-off method, following by annealing in a stream of 200 sccm (H_2_/N_2_ = 2:3) at 450 ºC for 15 minutes.

References

1. M. Dieterle, G. Mestl, Phys. Chem. Chem. Phys. **2002**, 4, 822.
2. M. A. Camacho-López, L. Escobar-Alarcón, M. Picquart, R. Arroyo, G. Córdoba, E. Haro-Poniatowski, Opt. Mater. **2011**, 33, 480.
3. H. B. Yang, X. Li, A. J. Wang, Y. Wang, Y. Y. Chen, Chinese Journal of Catalysis **2014**, 35, 140.
4. S. H. Lee, M. J. Seong, C. E. Tracy, A. Mascarenhas, J. R. Pitts, S. K. Deb, Solid State Ionics **2002**, 147, 129.
5. M, Kang,; Y. AKim, J. M, Yun, D. Y. Khim, J. H. Kim, Y. Y. Noh, K. J. Baeg, D. Y. Kim, Nanoscale **2014,** 6, 12315.
6. M. S. Choi, G. H. Lee, Y. J. Yu, D. Y. Lee, S. H. Lee, P. Kim, J. Hone, W. J. Yoo, Nat. Commun. **2013**, 4,1624.
7. E, Z. Zhang, W. Y. Wang, C. Zhang, Y. B. Jin, G. D.Zhu, Q. Q. Sun, D. W. Zhang, P. Zhou, F. X. Xiu, ACSNANO **2015**, 9, 612.
8. C. Y. Liu, A. Bard, J. Chem. Mater. **1998**, 10, 840.
